# Targeting the Host for New Therapeutic Perspectives in Hepatitis D

**DOI:** 10.3390/jcm9010222

**Published:** 2020-01-14

**Authors:** Vincent Turon-Lagot, Antonio Saviano, Catherine Schuster, Thomas F. Baumert, Eloi R. Verrier

**Affiliations:** 1 Inserm, Institut de Recherche sur les Maladies Virales et Hépatiques UMR_S1110, Université de Strasbourg, F-67000 Strasbourg, France; vincent.turon-lagot@etu.unistra.fr (V.T.-L.); saviano@unistra.fr (A.S.); catherine.schuster@unistra.fr (C.S.); thomas.baumert@unistra.fr (T.F.B.); 2 Institut Hospitalo-Universitaire, Pôle Hépato-Digestif, Nouvel Hôpital Civil, F-67000 Strasbourg, France

**Keywords:** Hepatitis D, antiviral strategy, host factors, liver disease

## Abstract

Hepatitis D virus (HDV) is a small satellite virus of hepatitis B virus (HBV) requiring HBV infection to complete its life cycle. It has been recently estimated that 13% of chronic HBV infected patients (60 million) are co-infected with HDV. Chronic hepatitis D is the most severe form of viral hepatitis with the highest risk to develop cirrhosis and liver cancer. Current treatment is based on pegylated-interferon-alpha which rarely controls HDV infection and is complicated by serious side effects. The development of novel antiviral strategies based on host targeting agents has shown promising results in phase I/II clinical trials. This review summarizes HDV molecular virology and physiopathology as well as new therapeutic approaches targeting HDV host factors.

## 1. Introduction 

Chronic hepatitis D is the most severe form of chronic viral hepatitis. It is caused by hepatitis D/delta virus (HDV), the smallest virus infecting mammals [1], which was first described in 1977 as a new hepatitis B virus (HBV)- associated antigen [2]. HDV is a small circular positive single stranded RNA virus, satellite of HBV relying on HBV surface protein HBsAg expression to produce new infectious particles [1,3,4]. HDV co- or super-infection in HBV chronically infected patients accelerates and worsens the progression of liver disease and triples the risk of developing hepatocellular carcinoma (HCC) [5,6]. HDV infection has been largely underestimated for the past decades. It was previously estimated that around 5% of chronic hepatitis B (CHB) patients were co-infected with HDV [7]. Recently, two different meta-analysis studies reevaluated this estimation at 13–14% of CHB patients, corresponding to 50–60 M people worldwide [7,8]. This prevalence corresponds to 0.8% of the general population. The previous underestimation can be explained by: (i) the absence of systematic screening of HDV in HBsAg positive patients; (ii) the recent improvement of HDV detection tools, leading to an increase in the number of diagnosed patients [9,10]. HDV infected patients are not equally distributed around the globe. Some regions are more affected by HDV infections, such as the Mediterranean basin, North and Central Africa, Russia and South-East Asia [7]. Some countries exhibit particularly high HDV prevalence such as Tunisia (15.33%), Mongolia (8.31%) and Nigeria (5.04%) [8].

Despite a very efficient HBV vaccine protecting against HDV infection, HBV and HDV infections are still on the rise worldwide. Current pegylated (PEG)-interferon (IFN)-alpha based therapy clears HDV infection in only 30% of the patients [11,12,13] and there is currently no other treatment that effectively controls HDV infection. Nevertheless, new therapeutic approaches are under preclinical and clinical development with some of them showing promising results.

## 2. HDV Life Cycle

**Figure 1:**
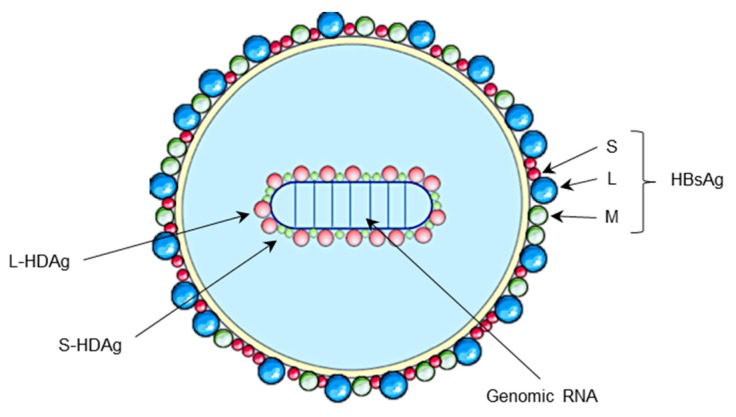
Hepatitis D virus (HDV) structure. (A) Schematic representation of HDV viral particle. HDV virion contains an envelope derived from the endoplasmic reticulum, in which are embedded the three forms (S, M and L) of hepatitis B virus (HBV) envelope protein, HBs antigen (HBsAg). HDV genome is a circular single stranded RNA of negative polarity associated to the two forms of delta antigen (L-HDAg and S-HDAg) forming a ribonucleoproteic complex.

HDV is characterized by a positive single-stranded circular RNA genome of around 1700 nucleotides size [14]. Interestingly, HDV RNA genome is predicted to have around 74% of paired bases giving it a rod-like structure [15]. It encodes only one protein, the delta antigen HDAg, expressed in two forms: the small form (S-HDAg) and the large form (L-HDAg) [1,16]. Both forms of HDAg have no enzymatic function and are associated with the HDV genome forming a ribonucleoprotein (RNP). During the life cycle, RNP is enveloped by HBV surface antigens (HBsAg) (Figure 1) [3,17]. Therefore, HDV complete life cycle depends on HBV infection and the expression of HBV envelop proteins. Importantly, HBV infection seems to be critical only for HDV particles assembly and egress but is not involved in other steps of HDV life cycle. Indeed, in vitro culture systems allow HDV replication and particle production in cells expressing only HDV genome and HBsAg without HBV replication [18,19]. This explains why antiviral nucleot(s)ide treatments, highly effective in controlling HBV replication, have no effect on HDV infection and pathogenesis [20].

**Figure 2:**
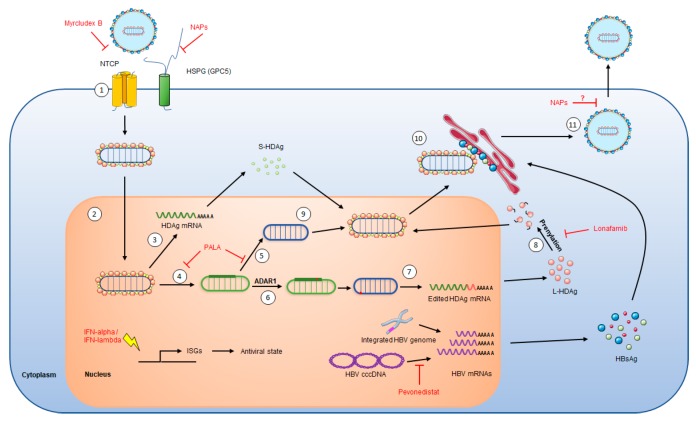
HDV life cycle. (1) HDV life cycle starts with HDV virions attachment to heparan sulfate proteoglycans (HSPG), including Glypican 5 (GPC5), at the hepatocyte surface. L-HBsAg pre-S1 region then binds to HBV/HDV specific receptor, the bile acid transporter NTCP. Viral particle enters the cell through endocytosis and viral RNP is freed in the cytoplasm. (2) Both forms of HDAg contain a nuclear localization signal that induces viral RNP translocation to the nucleus. (3) In the nucleus, HDAg mRNA transcription is done by RNA polymerase II. HDAg mRNA is then exported in the cytoplasm where it is translated to produce the small form of HDAg (S-HDAg). (4) During the first step of replication, HDV genomic RNA serves as a template for antigenomic RNA production, probably done by RNA polymerase I. (5) Antigenomic RNA is recognized by RNA polymerase II to produce new genomic RNAs. (6) Antigenomic RNA is edited by ADAR1 enzyme, suppressing S-HDAg stop codon. (7) Edited antigenomic RNA is replicated into genomic RNA, then inducing the transcription of edited HDAg mRNA that is exported in the cytoplasm where it leads to the production of the large form of HDAg (L-HDAg). (8) L-HDAg contains a prenylation site that is farnesylated by a cellular farnesyltransferase before being translocated to the nucleus. (9) Both forms of HDAg interact with newly synthesized genomic RNA to form new viral ribonucleoproteins (RNP) that are exported to the cytoplasm. (10) Viral RNPs interact, through their farnesylated cystein in L-HDAg, with the cytosolic part of HBsAg at the endoplasmic reticulum surface, thus inducing their envelopment. (11) HDV virions are then secreted form the infected cell. The different steps targeted by antiviral treatments are indicated. Represented cell is also infected by HBV, indicated by its cccDNA or its integrated genome, but its life cycle is not depicted.

**Figure 3:**
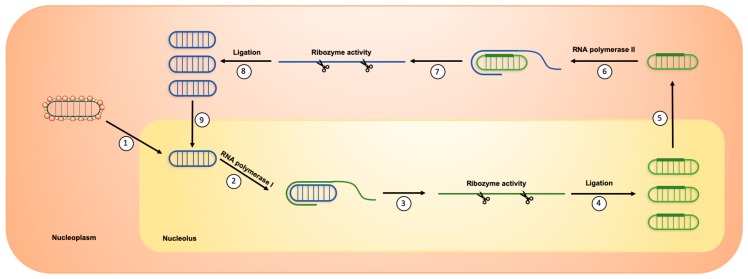
HDV replication. (1) HDV genome is translocated in the nucleolus. (2) It is then recognized by RNA polymerase I to produce concatemers of linear antigenomic RNAs through a rolling circle mechanism. (3) Ribozyme activity induced the cleavage of antigenomic RNA concatemers in antigenomic RNA monomers. (4) Linear antigenomic RNAs are circularized through an unknown ligation process. (5) Antigenomic RNAs are translocated in the nucleoplasm. (6) They are then recognized by RNA polymerase II to produce concatemers of linear genomic RNAs through a rolling circle mechanism. (7) Ribozyme activity induces the cleavage of genomic RNA concatemers into linear genomic RNA monomers. (8) Linear genomic RNAs are then circularized through an unknown ligation process. (9) Newly synthesized HDV genomic RNAs can be translocated again in the nucleolus for a new round of replication.

HDV tropism is restricted to hepatocytes, most likely due to HBsAg specific interaction with NTCP (sodium taurocholate co-transporting polypeptide), the receptor for HBV/HDV at the surface of hepatocytes [21,22]. HDV virions attach to heparan sulfate proteoglycans (HSPG), including GPC5, on the surface of the hepatocyte [23,24,25] and then specifically bind its receptor NTCP (Figure 2) [21,26]. HDV is then internalized through endocytosis and the viral RNP is released in the cytoplasm. Both forms of HDAg contain a nuclear localization signal (NLS) that addresses the viral RNP to the nucleus [27,28]. As the viral genome encodes only one structural protein, HDV replication fully relies on host polymerases. In the nucleus, RNA polymerase II usually employs DNA as a transcription template. However, HDV RNA seems to be recognized by RNA polymerase II, probably through its rod-like structure and interaction with S-HDAg and other cellular proteins, to be used as a template for HDAg mRNA transcription [29]. Newly synthesized S-HDAg then promotes viral replication that starts with the viral genome being transcribed by cellular RNA polymerase II to form viral anti-genomes of negative polarity through a rolling circle mechanism (Figure 3) [30,31]. However, former studies found that HDV anti-genome is synthesized by RNA polymerase I [29,32] and that HDAg mRNA is transcribed in the nucleoplasm and HDV anti-genome is synthesized in nucleoli [33,34]. Linear polymers of HDV anti-genomes are produced and self-cleaved through the ribozyme activity of the anti-genome. Single copies of anti-genome are then circularized. The newly synthesized anti-genome serves as a template for the production of *de novo* HDV genome copies by RNA polymerase II, through a similar rolling-circle mechanism (Figure 3) [31,32]. Recently, a genetic screen uncovered CAD, a protein involved in the first steps of uridine synthesis, as a key host factor for HDV replication affecting genomic and anti-genomic forms of viral RNAs [35]. Viral anti-genome can be edited by the cellular protein ADAR1, which induces an adenosine to inosine transformation in HDAg stop codon (Figure 2) [36,37]. This will further lead to the transcription of edited HDAg mRNA that will be translated into the large form of HDAg. In the cytoplasm, L-HDAg is farnesylated by a cellular protein [38,39] and the modified HDAg is translocated in the nucleus, promoting viral morphogenesis by inhibiting viral replication [40]. Newly synthesized HDV genomes associate with both forms of HDAg to form new viral RNPs that are exported from the nucleus via the TAP/Aly pathway [41] through the nuclear export signal (NES) located in the C-terminal part of L-HDAg [42]. In the cytoplasm, the viral RNP is recruited to the endoplasmic reticulum following interaction between the farnesylated L-HDAg and the cytosolic part of HBsAg [43]. This interaction induces HDV RNP envelopment and secretion from the infected cell through unknown mechanisms.

## 3. Physiopathology

**Figure 4:**
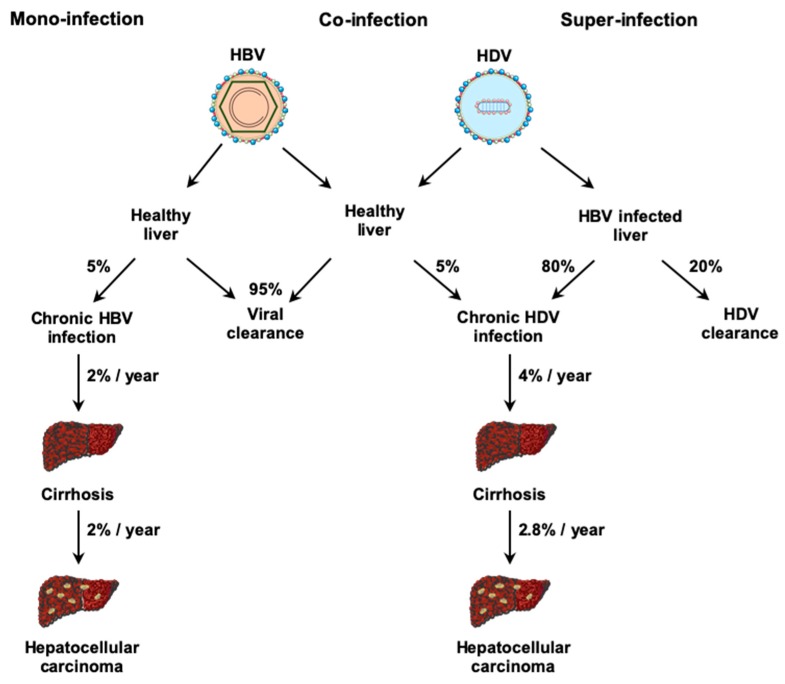
Natural history of HBV mono-infection and HDV co- and super-infection.

HDV can infect the liver by two different ways: in co-infection with HBV or in super-infection in chronic HBV (CHB) patients [44]. Whether HDV infection occurs in a co-infection or super-infection, the risk of fulminant hepatitis is increased compared to other viral hepatitis [45,46]. During co-infection, HDV is cleared by the immune system in 95% of adult patients such as HBV mono-infection (Figure 4) [44]. The ability of the immune system to efficiently eliminate HDV leads to a greater risk of fulminant hepatitis and liver failure [47,48]. In patients with a CHB, HDV super-infection is responsible for acute hepatitis [49] and induces general symptoms (e.g., fatigue, anorexia or nausea), elevation of hepatic inflammation markers such as serum ALT [50] and deterioration of liver function with risk of liver decompensation [49]. HDV super-infection leads to a chronic infection in 80% of CHB patients [44]. Chronic hepatitis D (CHD) patients present a threefold higher risk to develop cirrhosis [45] and HCC [6] than CHB patients. An Italian retrospective study followed 299 HDV infected patients for 28 years. During this period, 46 patients developed HCC, representing an annual rate of 2.8% [51]. Interestingly, HDV infection usually leads to a decrease in HBV replication, even though this effect may temporally vary [52,53]. However, when HBV is replicating, HBV-DNA load correlates with inflammatory activity and liver disease severity [54]. 

HBV is a stealth virus as it does not induce a strong innate immune response in vitro and in infected mice [55,56]. In the same way, HBV/HDV co-infection does not induce significant transcriptional modulations in primary hepatocyte cultures [57] even though HDV infection activates IFN-beta and IFN-gamma expression and induces a strong innate immune response in a cell culture model [58] and in a humanized mouse model [59]. The production of these antiviral and inflammatory cytokines has no effect on HDV replication but induces liver damage as a side effect in the attempt of clearing the virus. In vitro, HDAg repress the activity of two HBV promoters and L-HDAg activates *MX1* an interferon-induced antiviral protein [60]. The innate immunity activation in HDV infected hepatocytes and the partial inhibition of HBV transcription by HDAg could explain (i) how HDV directly and indirectly dampens HBV replication and (ii) how HBV/HDV co-infection accelerates and worsens liver disease compared to HBV mono-infection. 

In addition to HBV, HDV may interfere with another hepatotropic virus, hepatitis C virus, in patients co-infected with those three viruses. In Mongolia, a study on patients having either chronic hepatitis, liver cirrhosis or HCC showed that 30% of them were co-infected with the three viruses [61]. The molecular perturbations and pathogenesis outcomes seem unclear. Some studies report that HDV becomes the dominant virus and suppresses HBV and HCV replication [62]. Chronic patients with triple infection show a higher progression to cirrhosis and a higher risk to develop HCC compared to HBV/HDV patients. Moreover, during acute phase of super infection there is an increased risk of fulminant hepatitis. It was recently shown in vitro that HDV viral particles could be enveloped by HCV glycoproteins instead of HBsAg. It would be interesting to see in those patients if there are HDV/HCV co-infected hepatocytes and if infectious HDV/HCV particles are produced [63]. This could also give hints if HDV perturbations of HCV replication are mediated by a direct or an indirect mechanism.

## 4. Therapeutics: Past and Future

### 4.1. Current Treatment: Pegylated Interferon Alpha

The only treatment currently available for the clinical practice is PEG-IFN-alpha. Only 30% of HDV patients respond to PEG-IFN-alpha and rarely achieve viral clearance [11,12,13]. Furthermore, PEG-IFN-alpha is responsible for many serious side effects including flu-like symptoms, nausea, insomnia and depression, frequently leading to treatment discontinuation [64,65,66]. The other challenge is that, to be successful, a treatment must inhibit both HDV replication and HBsAg production, since the inhibition of HDV replication alone is often followed by a relapse in HDV replication after the end of treatment. Unfortunately, nucleot(s)ides analogs treatment used against HBV infection are ineffective against HDV replication because they do not reduce HBsAg production [20]. The lack of viral proteins expressed by HDV strongly limits the development of direct-acting antivirals. The only HDV enzymatic activity relies on the ribozyme. However, ribozyme inhibitors exhibited a marked toxicity in vitro and have therefore not been extensively studied for HDV treatment [67]. 

In this context, host targeting agents (HTAs) represent attractive therapeutic strategies, by targeting host proteins whose function is required for virus infection. Some of these drugs are already used in other chronic viral infections, such as Maraviroc, an antagonist of CCR5 that inhibits HIV entry [68]. HTAs reduce the risk of viral resistance to treatment, especially against RNA viruses that are known to mutate and adapt rapidly [69,70]. Since HDV largely relies on host proteins to fulfill each step from entry to viral particles secretion, the identification of these host factors would provide new cellular targets for viral cure. Even though the molecular interactions between HDV and hepatocyte host factors are still largely unknown, several HTAs are currently tested in clinical trials.

### 4.2. New Therapeutic Agents in Clinical Trials

#### 4.2.1. Interferon-Lambda, a Better Tolerated Immunomodulator 

Interferon-lambda (IFN-lambda) is the type III interferon and, upon binding to its receptor, induces a signaling cascade similar to the one induced by IFN-alpha [71]. Contrary to IFN-alpha receptor, IFN-lambda receptor is not ubiquitously expressed therefore leading to limited side effects. A recent study called LIMT (for Lambda Interferon MonoTherapy) HDV investigated the safety and efficacy of pegylated IFN-lambda (PEG-IFN-lambda) monotherapy in chronic HDV infected patients from Pakistan, Israel and New-Zealand [72]. In this trial, 33 HDV patients receiving an anti-HBV nucleos(t)ide analog were treated with either PEG-IFN-lambda 180 µg (*n* = 14) or 120 µg (*n* = 19) by weekly subcutaneous injections for 48 weeks. After treatment, the patients were followed up for 24 weeks and 7 out of 14 patients (50%) from the 180 µg group exhibited at least a 2-log decline in HDV RNA and 5 (36%) of them were below the quantification limit. Side effects reported are flu-like symptoms and elevated transaminase levels. However, these side effects appear to be milder than the side effects caused by PEG-IFN-alpha. In the Pakistani cohort, some cases of jaundice and bilirubin elevation were reported. Overall, PEG-IFN-lambda showed better results and less side effects than treatment with PEG-IFN-alpha. Even though the decrease in HDV RNA induced in monotherapy is observed in only 50% of patients, PEG-IFN-lambda seems to be a good alternative to IFN-alpha treatment.

#### 4.2.2. Myrcludex B, an Entry Inhibitor

HBV and HDV share the same envelop and thus the same entry pathway in hepatocytes, involving the binding to the NTCP receptor (Figure 2) [22]. NTCP interacts with the N-terminal region of HBsAg preS1 domain. Even before the identification of NTCP as HBV/HDV receptor, it was already known that myristoylated peptides derived from the 78 aa of the preS1 domain inhibits HBV infection in vitro and in vivo [73,74]. Following discovery of NTCP as the HBV/HDV receptor, it has been confirmed that synthetic preS1 derived peptides specifically bind to NTCP therefore inhibiting HBV entry into hepatocytes [21,75]. One of these peptides containing 47 aa is now commercialized with the name Myrcludex B (MyrB) or Bulevirtide [76] and has been extensively tested in clinical trials. 

The final results from the first phase II clinical trial involving MyrB treatment in combination with tenofovir (TDF) were recently released [77]. In this trial 120 chronic HDV patients were split in four groups. Three groups were treated for 24 weeks with TDF 245 mg/day and different doses of MyrB: 2 (A), 5 (B) or 10 (C) mg administered daily by subcutaneous injection. The last group (D) received TDF 245 mg/day alone for 24 weeks. After this period, all groups received an additional TDF treatment for 24 weeks. At the end of treatment, primary endpoint was reached for 46.4% (A), 46.8% (B), 76.6% (C) and 3.3% (D) of patients. Alas, at 12 weeks follow up, HDV relapse was observed in 60% (A), 80% (B) and 83% (C) of responder patients even though HDV RNA median was still lower than baseline. Giving the high number of HDV relapse, a phase II clinical trial assessing the efficacy of MyrB in combination with PEG-IFN-alpha was performed [78]. Sixty patients were split in four groups and were treated for 48 weeks with either: (A) 180 µg PEG-IFN-alpha, (B) 2 mg MyrB + 180 µg PEG-IFN-alpha, (C) 5 mg MyrB + 180 µg PEG-IFN-alpha or (D) 2 mg MyrB (Table 1). MyrB was administered once daily and PEG-IFN-alpha once a week, both by subcutaneous injections. Briefly, monotherapy with either PEG-IFN-alpha (A) or 2 mg MyrB (D) exhibited a poor response to treatment with only two patients out of 15 (13%) in each group having undetectable HDV RNA at the end of the treatment (EOT). All of them relapsed within the follow-up period. In group B, 9/15 (60%) of patients exhibited undetectable HDV RNA at EOT and only one relapse in 24 weeks after EOT (Table 1). Furthermore, this group is the only one that had a decrease in HBsAg production with 6/15 (40%) of patients having at least 1 log decrease in HBsAg compared to baseline at the end of the follow-up period. MyrB frequently induces bile acid increase that can lead to mild/moderate side effects but the safety in cirrhotic patients has to be demonstrated.

Overall, this treatment does not seem really suitable for monotherapy since it does not inhibit HBsAg production by itself [79]. Combination treatments seem to be more effective at inhibiting both HDV replication and HBsAg production [77,78]. It is to note that in France, Bulevirtide (commercial name of MyrB) recently got an exceptional temporary authorization of use in patients having cirrhosis or severe fibrosis [80], showing the growing interest in this treatment. 

#### 4.2.3. Lonafarnib, a Morphogenesis Inhibitor

During the HDV life cycle, the C-terminal domain of L-HDAg is farnesylated by a cellular farnesyltransferase (Figure 2). This post-translational modification is required for L-HDAg interaction with HBsAg at the endoplasmic reticulum, thus inducing virions envelopment [43]. Lonafarnib is a farnesyltransferase inhibitor that was first investigated as a treatment for Hutchinson–Gilford progeria syndrome [81]. Lonafarnib (LNF) has been investigated in combination with ritonavir (RTV) through four different phase II studies called LOWR-HDV [82,83,84,85]. RTV does not have an antiviral effect but is an inhibitor of LNF metabolism and thus increases its availability, stability and efficacy at lower doses [83]. LOWR-HDV-4 was a dose escalation study and enrolled 15 patients. All patients first started with 50 mg LNF and 100 mg RTV administered twice a day. After four weeks, if the treatment was well tolerated LNF dose was increased to 75 mg and after two more weeks it was increased to 100 mg. Overall, patients were treated for 24 weeks followed by 24 weeks post-treatment follow-up [85]. Highest LFN dose could be administered to 10/15 patients (66%) but only five remained at this dose until end of treatment. At EOT, only one patient became PCR-negative and one had HDV RNA below limit of detection. The latter remained below limit of detection at eight weeks follow up. 

Recently, another phase IIa study released interim results [86]. In this study called LIFT, 26 patients were treated with 50 mg LNF and 100 mg RTV twice daily and weekly subcutaneous injection of 180 mcg pegylated-interferon-lambda-1a (LMB) for 24 weeks (Table 1). Data at 24 weeks of treatment were available only for 19 patients. Treatment dose was reduced in three patients and four patients discontinued treatment. At EOT, 18/19 patients (95%) exhibited a greater than 2 log HDV RNA decrease and 10/19 (53%) showed undetectable HDV RNA level. These preliminary results suggest a greater efficacy of combination treatment, with mild to moderate side effects. 

Finally, LNF will be studied in a phase III clinical trial called D-LIVR (ClinicalTrials.gov Identifier: NCT03719313) that started in December 2018. In this clinical trial, patients will be treated for 48 weeks with 50 mg LNF and 100 mg RTV with or without weekly injection of 180 mg PEG-IFN-alpha-2a. The estimated completion date for this clinical trial is April 2021.

#### 4.2.4. Nucleic Acid Polymers, Inhibitor of HBsAg Secretion

Nucleic acid polymers (NAPs) are phosphothiorated oligonucleotides having a great in vivo denaturation and degradation resistance. Previous data reveal a broad-spectrum antiviral activity including inhibition of hepatitis C virus entry [87] and infection by several herpesviruses’ infection [88]. Different NAPs exhibit an antiviral activity against HDV even though their mechanism of action is not fully understood. It is supposed that NAPs could act at different levels against HDV infection, inhibiting viral entry [89] or HBsAg secretion [90]. Interestingly, their antiviral activity does not seem to rely on their sequence but rather depends on their size and hydrophobicity [90]. First, two different NAPs, called REP2055 and REP2139, were studied in chronic hepatitis B patients [91]. Both compounds showed a strong antiviral activity, sometimes accompanied with seroconversion. REP2139 being more tolerated than REP2055, it has been chosen for further clinical trials. REP2139 safety and efficacy against HBV and HDV in co-infected patients was assessed in the REP-301 clinical trial. In this trial, 12 patients were treated weekly by intravenous injection of 500 mg REP2139 for 15 weeks, then with 250 mg REP2139 combined with subcutaneous injection of 180 µg PEG-IFN-alpha-2a for 15 weeks, followed by a final treatment of weekly subcutaneous injection of 180 µg PEG-IFN-alpha-2a for 33 weeks (Table 1). All the 12 patients experienced side effects including neutropenia (67%), thrombocytopenia (83%) or increased alanine aminotransferase (ALT) levels (42%). Furthermore, four patients (33%) had serious side effects including elevated alanine and aspartate aminotransferase concentrations and strong thrombocytopenia, however all of them were attributed to PEG-IFN-alpha-2a treatment [92]. During treatment, 11 patients (92%) became HDV RNA negative and nine of them (75%) remained negative at the end of treatment. Recently, results from a long-term follow-up of 2.5–3 years reported that the nine patients that previously were HDV RNA negative still had more than 2 log HDV RNA reduction from baseline and seven of them (58%) were still HDV RNA negative (Table 1) [93]. Regarding those seven patients, all of them had asymptomatic transaminase flare while having HBsAg concentration lower than 1 IU/mL during therapy and 4 of them (57%) had a functional HBV cure with HBsAg below the limit of detection, undetectable HBV DNA and normal ALT.

Functional studies showed that antiviral effect of REP2139 can be active through two different mechanisms of action. First, it inhibits HBsAg secretion, thus inhibiting HBV and HDV viral particle envelopment and secretion. It can also interact with both forms of HDAg, interaction with S-HDAg could inhibit HDV replication and interaction with L-HDAg could inhibit HDV RNP assembly (Figure 2) [94]. Overall, REP2139 showed a great antiviral activity against both HBV and HDV, but these results need to be confirmed on larger cohorts of patients.

### 4.3. In Vitro Studies

#### 4.3.1. PALA, Inhibitor of HDV Replication

This year, a genetic screen identified pyrimidine metabolism pathway as important for HDV replication [35]. Specifically, in vitro experiments showed that silencing or knock-out of CAD, an enzyme involved in the first three steps of uridine biosynthesis, induced a strong decrease in HDV replication. Sparfosic acid or PALA (N-(phosphonoacetyl)-l-aspartic acid) is a specific inhibitor of CAD activity [97]. Treatment of HDV infected cells with PALA strongly decreased HDV replication without major toxicity and this effect could be reversed by media complementation with uridine showing the specificity of action of PALA (Figure 2) [35]. Furthermore, PALA treatment has no effect on HBV replication. It is to note that PALA was already investigated in a phase II clinical trial for patients with advanced gastric carcinoma and that no major toxicity was observed [98]. Therefore, PALA could be used in combination therapy in co-infected patients.

#### 4.3.2. Pevonedistat, Inhibitor of HBV Transcription

In addition to the inhibition of the HDV life cycle per se, HBsAg production has to be targeted to avoid HDV relapse after treatment [99]. In this context, one strategy consists in a combination treatment including anti-HDV agents and inhibitors of HBV transcription. Neddylation is an ubiquitylation-like process inducing neuronal precursor cell-expressed developmentally down-regulated protein 8 (NEDD-8) combination with a specific substrate usually inducing activation or increasing stability [100]. The first discovered neddylation substrates are Cullin proteins. Their neddylation induces specific ubiquitylation and proteasomal degradation of protein substrates. It was recently shown that HBV regulatory protein X (HBx) uses this neddylation process to target the structural maintenance of chromosomes 5/6 (Smc5/6) protein and induce its ubiquitylation and degradation, therefore enhancing HBV transcription from cccDNA [101,102]. Pevonedistat (or MLN4924) is a specific inhibitor of NEDD8 Activating Enzyme E1 Subunit 1 (NAE1), the first protein involved in NEDD8 catalyze [100] and has recently been studied in a phase Ib clinical trial in patients with solid tumors [95]. A recent in vitro study showed that pevonedistat treatment in HBV-infected cells or PHH could effectively restore Smc5/6 protein level, thus inducing HBV transcription inhibition (Figure 2) [96]. The effect on HBV transcription was induced at micromolar concentration without any observed toxicity. Pevonedistat inducing an inhibition of HBsAg production, it could be used in combination with HDV replication inhibitors. The combination could indeed induce a real elimination of HDV infected cells and clearance of the virus.

## 5. Conclusions

HDV is a very peculiar virus responsible for the most severe form of viral hepatitis. There is, to date, no treatment able to cure HDV infection, thus representing a major health issue. However, two treatments, i.e., MyrB and LNF, have completed phase II clinical studies and LNF already started a phase III clinical study. Bulevirtide (MyrB) recently got an exceptional temporary authorization of use in France for patients having cirrhosis or severe fibrosis [80]. Overall, the new compounds for the treatment of HDV show higher effects when combined with PEG-IFN and when a strong reduction or clearance of HBsAg is obtained. The complexity of the HDV/HBV infection and the virus-host interactions should be considered in the therapeutic approach of this disease. PEG-IFN-alpha treatment is still a mainstay and combination therapies targeting also HBV have the highest chance to success.

More virus-host interactions studies will certainly lead to the discovery of new HDV host factors and therapeutics. Therefore, effort must be maintained in this field. HDV infection is still underdiagnosed even though diagnostic tools are becoming faster and cheaper. More efforts should be put to fill this gap, vaccinate for HBV and screen for HDV infection all the HBV infected patients. There is no use of developing antiviral molecules if patients are not aware of their status and cannot be treated. Improvements in all these directions will hopefully lead to the eradication of this major hepatic virus.

## Figures and Tables

**Table 1:** Antiviral molecules in clinical trial. MyrB: myrcludex B; PEG-IFNA: pegylated-interferon-alpha-2a; LNF: lonafarnib; RTV: ritonavir; LMB: pegylated-interferon-lambda; CT: clinical trial; LLOQ: lower limit of quantification; EOT: end of treatment; FU: follow-up; SSE: serious side effect; ND: no data.

	Cellular Target/Step of HDV Life Cycle	Current Clinical Trial Step	Posology	EOT	FU (24w)	Drawbacks	References
MyrB + PEG-IFNA	NTCP/Entry inhibitor	Phase III	Phase II CT:	LLOQ: 15/30 (50%)	LLOQ: 12/30 (40%)	3 relapses at FU (24 weeks); SSE: 5	[78]
			2 or 5 mg MyrB daily + 180 µg IFNA weekly (48 weeks)				
LNF + RTV + LMB	Farnesylation/Assembly inhibitor	Phase III	Phase II CT:	>2log decrease: 18/19 (95%)	ND	ALT flares at EOT	[86]
			50 mg LNF + 100 mg RTV twice a day	LLOQ: 10/19 (53%)			
			180 mcg LMB weekly				
REP2139	?/HBsAg secretion inhibitor	Phase II	500 mg weekly (15 weeks), 250 mg + 180 µg IFNA (15 weeks), 180 µg IFNA (33 weeks)	>2log decrease: 9/11 (82%)	>2log decrease: 9/11 (82%)	SSE in 4/11 patients (33%)	[92,93]
				LLOQ: 9/11 (82%)	LLOQ: 7/11 (63%)		
PALA	CAD/HDV replication	Preclinical study	100 µM in cultured PHH without toxicity	ND	ND	Safety in an animal model not assessed yet	[35]
Pevonedistat	NAE1/HBV transcription	Preclinical study	1 µM in cultured PHH without toxicity	ND	ND	Mild to strong SE observed in phase Ib CT	[95,96]
